# Hormonal Therapy Resistance and Breast Cancer: Involvement of Adipocytes and Leptin

**DOI:** 10.3390/nu11122839

**Published:** 2019-11-20

**Authors:** Laetitia Delort, Lauriane Bougaret, Juliette Cholet, Marion Vermerie, Hermine Billard, Caroline Decombat, Céline Bourgne, Marc Berger, Charles Dumontet, Florence Caldefie-Chezet

**Affiliations:** 1 INRA, UNH, Unité de Nutrition Humaine, CRNH Auvergne, Université Clermont Auvergne, F-63000 Clermont-Ferrand, France; laurianebougaret@hotmail.fr (L.B.); Juliette.CHOLET@uca.fr (J.C.); Marion.VERMERIE@uca.fr (M.V.); hermine.billard@uca.fr (H.B.); Caroline.DECOMBAT@uca.fr (C.D.); florence.caldefie-chezet@uca.fr (F.C.-C.); 2 Service d’Hématologie Biologique, CHU Estaing, F-63000 Clermont-Ferrand, France; cbourgne@chu-clermontferrand.fr (C.B.); mberger@chu-clermontferrand.fr (M.B.); 3 Université Lyon 1, INSERM U1052, CNRS 5286, Cancer Research Center of Lyon, 69008 Lyon, France; charles.dumontet@chu-lyon.fr

**Keywords:** breast cancer, adipose secretome, leptin, hormonal therapy resistance, cancer stem cells

## Abstract

Obesity, a recognized risk factor for breast cancer in postmenopausal women, is associated with higher mortality rates regardless of menopausal status, which could in part be explained by therapeutic escape. Indeed, adipose microenvironment has been described to influence the efficiency of chemo- and hormonal therapies. Residual cancer stem cells could also have a key role in this process. To understand the mechanisms involved in the reduced efficacy of hormonal therapy on breast cancer cells in the presence of adipose secretome, human adipose stem cells (hMAD cell line) differentiated into mature adipocytes were co-cultured with mammary breast cancer cells and treated with hormonal therapies (tamoxifen, fulvestrant). Proliferation and apoptosis were measured (fluorescence test, impedancemetry, cytometry) and the gene expression profile was evaluated. Cancer stem cells were isolated from mammospheres made from MCF-7. The impact of chemo- and hormonal therapies and leptin was evaluated in this population. hMAD-differentiated mature adipocytes and their secretions were able to increase mammary cancer cell proliferation and to suppress the antiproliferative effect of tamoxifen, confirming previous data and validating our model. Apoptosis and cell cycle did not seem to be involved in this process. The evaluation of gene expression profiles suggested that STAT3 could be a possible target. On the contrary, leptin did not seem to be involved. The study of isolated cancer stem cells revealed that their proliferation was stimulated in the presence of anticancer therapies (tamoxifen, fulvestrant, doxorubicine) and leptin. Our study confirmed the role of adipocytes and their secretome, but above all, the role of communication between adipose and cancer cells in interfering with the efficiency of hormonal therapy. Among the pathophysiological mechanisms involved, leptin does not seem to interfere with the estrogenic pathway but seems to promote the proliferation of cancer stem cells.

## 1. Introduction

Breast cancer is the most common cancer among women with 523,000 new cases, which represents 13.4% of all cancer cases in Europe and is the leading cause of death in women (138,000, 16.2%) [1]. Many epidemiological studies have confirmed the link between obesity and the development of cancers such as colon, prostate, and more recently, ovarian cancer. Overweight and obesity are associated with a higher risk of postmenopausal breast cancer (RR = 1.12, 95% confidence interval [95% CI] = 1.09–1.15) [1], larger tumor, positive lymph-node status, and reduced outcome regardless of menopausal status [2]. Furthermore, weight change during cancer treatment could also be associated with a poorer diagnosis [3]. Despite the accumulation of evidence linking obesity to the development of breast cancer, this factor is rarely taken into account and could be decisive in the implementation of individualized treatment for overweight patients.

The mechanisms by which obesity could interact with the development of cancer are complex and not fully understood. The importance of the mammary tumor microenvironment in the development, growth, and progression of cancer is widely recognized today. Interactions between the different cell types present in the adipose microenvironment are now described. This mammary adipose microenvironment is very heterogeneous and mainly consists of adipocytes (50% to 80% of the vascular fraction) as well as a set of cell types forming the stromal-vascular fraction and containing adipose stem cells, endothelial and immune cells, fibroblasts, as well as extracellular matrix (laminin, fibronectin, collagens, proteoglycans). Adipose tissue is now considered to be an endocrine organ capable of secreting soluble factors that can act on surrounding cells and on the composition of the extracellular matrix [4]. These are mainly growth factors, cytokines, adipokines, proteases, or vascular stimulation factors. Breast stroma can undergo phenotypic and functional changes to be active and provide a favorable environment for mammary tumor development [5].

Resistance of cancer cells to therapies has been proposed to explain the link between obesity and the higher mortality observed in breast cancer patients. Indeed, studies suggest such resistance for aromatase inhibitors [6] or neoadjuvant chemotherapies, such as anthracyclines and taxanes [7]. About 70% of breast cancers express estrogen receptors (ER+) and the most effective treatment is hormonal therapy which blocks estrogen activity. Current hormonal therapies are mainly tamoxifen (Tx), an estrogen receptor antagonist, fulvestrant (Fv), a pure anti-estrogen responsible for the downregulation and degradation of this receptor, and aromatase inhibitors such as anastrozole and letrozole [8]. Unfortunately, de novo or acquired resistance could be developed, leading to disease progression and increased mortality, which could be amplified in overweight women [9]. Some authors demonstrated that aromatase inhibitors may be less efficient than tamoxifen in overweight and obese patients [10]. Tx resistance seems to be multifactorial with, for example, the lack of ER function or disruption of signaling pathways (RTK, PI3K, NF-kB) [11].

The role of cancer stem cells (CSCs) in hormonal therapy resistance has also been investigated. CSCs have common properties with normal stem cells, including their ability to self-renew and differentiate [12], but also additional specific features, such as uncontrolled proliferation and partial or abnormal differentiation, contributing to increased tumorigenicity in component tumors, potentially driving metastases. CSCs, previously discovered in liquid tumors, were finally found in solid tumors such as breast cancer in which CSCs could represent 0.1% to 2% of all breast cancer cells [13]. Breast CSCs express a particular phenotype based on the expression of markers such as CD44^+^/CD24^−^, an enzymatic activity of aldehyde dehydrogenase (ALDH), an overexpression of transmembrane pumps (ATP-binding cassette, subfamily G, member 2 ABCG2) leading to the exclusion of Hoechst 33342 fluorescent probe and the characterization of a cell population called a “side-population” (SP) fraction [14]. These SP fraction may therefore be able to reject toxic drugs, indicating their involvement in cancer drug resistance. The role of CSCs in resistance to hormonal therapy has also been investigated. Tx is able to promote the CSCs survival in MCF-7, since a pretreatment with 4-hydroxytamoxifen (4-OH-Tx), a metabolite of Tx, raises their ability to form mammospheres [15].

Thus, a worse prognosis is observed in obese or overweight women treated with hormone therapy with Tx that is more effective than anti-aromatase inhibitors, the resistance mechanisms remaining unknown. So the aim of this study was to investigate (i) how obesity could interfere with hormonal therapies by evaluating the role of the adipose microenvironment in signaling pathways and (ii) the comportment of isolated mammary CSCs in the presence of hormonal therapy and adipokines.

## 2. Materials and Methods

### 2.1. Study of Breast Cancer Cells

#### 2.1.1. Cell Culture

*Breast cancer cells.* MCF-7 (estrogen-receptor positive [ER+]) and MDA-MB-231 (ER-) breast cancer cell lines were obtained from ATCC (Manassas, VA, USA). MCF-7 were cultured in RPMI1640 (Gibco, Thermo Fisher Scientific, Waltham, MA, USA) supplemented with insulin (0.04 UI/mL) (Sigma-Aldrich, Saint-Louis, MO, USA), fetal bovine serum (10%), L-glutamine (1%), and gentamycin (50 µg/mL) (Thermo Fisher Scientific). MDA-MB- 231 were grown in L15 (Thermo Fisher Scientific) supplemented with FBS (15%), L-glutamine (1%), and gentamycin (50 µg/mL). Cells were incubated at 37 °C following recommendations of ATCC in a humidified 95% air/5% CO_2_ environment for MCF-7 and 100% air for MDA-MB-231.

*Adipose cells.* The human multipotent adipose (hMAD) cell line is a generous gift from C. Dumontet (University Lyon 1, Lyon, France; INSERM U1052, CNRS 5286, Cancer Research Center of Lyon, Lyon, France). hMAD were differentiated into mature adipocytes (MA) [16]. For that, adipose cells were seeded at confluence (33,500 cells/cm^2^) in a differentiation medium which consisted of DMEM/F12 (1:1) supplemented with 10% FBS, hydrocortisone, insulin, adenine, EGF, T3, vitamin C, dexamethazon, roziglitazone, IBMX (only the first three days), gentamycin. The medium was replaced every two days. MA were obtained after 12 days of differentiation.

All the cells were under mycoplasma-free conditions (MycoAlertTM PLUS Mycoplasma detection Kit, Lonza, Basel, Switzerland).

#### 2.1.2. Influence of Adipose Secretome

To assess the specific role of adipose secretome, the proliferation of mammary cancer cells (MCF-7 and MDA-MB-231) cultured with conditioned media (CM) obtained from the culture of the MA was evaluated using the iCELLigence technology which allows automatic monitoring of cell adherence and proliferation in real-time. CM were collected after 48 h of culture of MA in DMEM/F12 supplemented with FBS (10%) and glutamine (1%). Before use, they were kept under nitrogen atmosphere at −80 °C. After 24 h of adhesion in the iCELLigence system, cells were exposed to CM (dilution 1:1 in fresh complete adipose cell media) and/or tamoxifen (Tx, IC_50_ = 10 µM, Sigma-Aldrich) for 72 h. The impedance value of each well was measured by the iCELLigence system every 10 min for 72 h and expressed as cell index (CI) values. Data for cell adherence were normalized at 24 h corresponding to the time of treatment to give a normalized cell index. Three independent experiments were conducted.

#### 2.1.3. Evaluation of Cell Cell Interactions

A system of co-cultures was used between breast cancer cells seeded at the bottom of wells and MA seeded in inserts, allowing to assess the interactions between the two cell types through a porous membrane (Transwell culture system, porosity 0.4µm).

For that, hMAD were seeded in inserts (Merck Millipore, Molsheim, France) and differentiated into MA for 12 days. At the end of the differentiation, MCF-7 or MDA-MB-231 were seeded (15,150 cells/cm^2^) at the bottom of the plate in DMEM/F12 medium supplemented with FBS (10%) and glutamine (1%). Twenty-four hours later, treatment with antiestrogen (Tx, IC_50_ = 10µM, or Fv, IC_50_ = 0.5nM, Sigma-Aldrich) and/or an anti-leptin antibody (0.5 µg/mL) (R&D Systems Europe, Lille, France) were added in the co-culture system.

The proliferation was measured after 72 h with the resazurin test (3 independent experiments) (Exw = 530 nm and Emw = 590 nm, Fluoroskan Ascent FL^®^, Thermo Fisher Scientific). Results were expressed as a percentage of cell growth ± ESM.

#### 2.1.4. Annexin V–FITC/PI Apoptosis Assay

MCF-7 mammary cancer cells cultured with CM and/or Tx (10 µM) for 72 h were washed with phosphate buffered saline (PBS), recovered by centrifugation at 1000 *g* for 5 min at room temperature (RT), and suspended in 40 µL of annexin V binding buffer (140 mM NaCl, 10 mM HEPES/NaOH, 2.5 mM CaCl_2_). The cell suspension was stained with 5 µL of annexin V-FITC and 5 µL of propidium iodide (PI), and incubated for 15 min at RT in the dark. After the addition of PBS and a centrifugation at 1000 *g* for 5 min at RT, cells were suspended in 50 μL of annexin V binding buffer. Stained cells were then analyzed on a Cellometer K2 image cytometer (Nexcelom Bioscience, Lawrence, MA, USA). Data were expressed as a percentage of live cells, % of cells in early or late apoptosis and % of necrotic cells. Experiments were realized three times.

#### 2.1.5. Quantitative Real-Time PCR (qPCR) Assays

Total RNA was extracted from MCF-7 mammary cells that had been co-cultured with MA and treated with Tx using Trizol (Invitrogen, Thermo Fisher Scientific). After the evaluation of the quantity and purity (NanoDrop 2000, Thermo Fisher Scientific), DNase treatment (DNase I Amplification grade, Invitrogen, Thermo Fisher Scientific) and cDNAs retrotranscription (HighCap cDNA RT Kit RNAse inhib, Invitrogen) were made according to the manufacturer’s recommendations. qPCR was performed on plates designed by Applied Biosystems (TaqMan^®^ Array 96 well Fast Plate, Custom format 32) using SDS7900HT automaton (Applied Biosystems, Thermo Fisher Scientific) with TaqMAN^®^ (Applied Biosystems, Thermo Fisher Scientific). The analysis was conducted on 32 genes (LEPR; LEP; ADIPOQ; ADIPOR1; ADIPOR2; ESR1; ESR2; PGR; CYP19; CDH1; MMP2; MMP9; IL6; TNF; IGF1; VEGFA; MYC; AKT1; PTEN; CTNNB1; TWIST1; BAX; BCL2; CCND1; MAPK1; MAPK3; MAPK8; TP53; STAT3) and 3 reference genes (18S; UBC; ACTB). Genes were considered significantly expressed and their transcript measurable if their corresponding Ct value was less than 35. The relative quantification method (RQ = 2^−ΔΔCT^) was used to calculate the relative gene expression of given samples with ΔΔCT = [ΔCT (sample1) − ΔCT (sample2)] and ΔCT = [CT (target gene) − geometric mean CT (reference genes)]. Paired t-tests were used to compare gene expression levels with at least 2 valid pairs of values. Control of the false discovery rate due to multiple testing was done according to the Benjamini Hochberg method for each comparison separately. Using ΔCT values, gene expression was plotted as a heatmap, paired with a two-way hierarchical cluster analysis (package “gplots”) in R version 3.5.0. To infer how gene expression covaried with the factors “Tx treatment” and “co-cultured MCF-7”, a multiple factor analysis (MFA) was carried out on ΔCT using the package “FactoMineR”.

### 2.2. Study of Cancer Stem Cells (CSCs)

#### 2.2.1. Mammosphere Formation

MCF-7 cells were seeded (8 × 10^3^ cells) in 96 wells in “Ultra Low Attachment Plates” (Corning, NY, USA) in DMEM/F12 without phenol red (Gibco, Thermo Fisher Scientific) supplemented with b-FGF (10 ng/mL; Merck Millipore, Darmstandt, Germany), EGF (20 ng/mL; Gibco), B27 (2%; Gibco), and gentamicin (50 mg/mL), in a 5% CO_2_-humidified incubator at 37 °C. After 7 days of culture, mammospheres were recovered by gentle pipetting, dissociated in accutase (Merck Millipore) for 30 min at 37 °C, reseeded and cultured in the same conditions until passage 3.

#### 2.2.2. Staining of Side-Population Cells (SP) by Flow Cytometry

SP staining was essentially performed as described by Goodell et al. [17]. Single cell suspensions of parental MCF-7 or mammospheres (10^6^ cells/mL), obtained after incubation in accutase (30 min), were incubated in DMEM/F12 (2% FBS, 5 mg/mL Hoechst 33342, 120 min, 37°; Sigma-Aldrich) in a dark water bath under agitation with or without ABC transporter inhibitor (50 µM Verapamil, Sigma-Aldrich). After incubation, cells were maintained at 4 °C to inhibit dye efflux.

#### 2.2.3. Phenotyping of SP Cells

Cells were incubated in cold PBS-2% FBS with directly-conjugated primary antibodies or isotype controls, i.e., CD44 FITC (10 µL per test) or CD24 PE (10 µL per test) (BD Pharmingen, San Jose, CA, USA) for 20 min at 4 °C in the dark according to the manufacturer’s instructions. PI (5 µg/mL) was added to discriminate dead cells, and cell suspensions were filtered through a 40 µm cell strainer (BD Biosciences, San Jose, CA, USA) just prior to analysis.

#### 2.2.4. Identification of ALDH-1-Expressing Cells

Cells were analyzed using an ALDEFLUOR detection kit (StemCell Technologies, Vancouver, BC, Canada) according to the manufacturer’s instructions. The activated BODIPYTM-aminoacetaldehyde (BAAA) is a fluorescent non-toxic substrate for ALDH that diffuses into viable cells. In the presence of ALDH1, BAAA is converted into BODIPYTM-minoacetate, which is retained inside the cells. The amount of fluorescence reaction is proportional to ALDH1 activity. A specific inhibitor of ALDH, diethylaminobenzaldehyde (DEAB), is used to control for background fluorescence.

#### 2.2.5. Cytometry Analysis, Cell Sorting

Viable cells were analyzed and isolated by flow cytometry (BD FACSAria SORP, BD Biosciences) using an 85 µm nozzle. Data was acquired at 4 °C using BD Diva 7.0 software. The cytometer was equipped with an UV laser (OPSL 3.6, 100 mW, Coherent). Hoechst Blue and Hoechst Red were detected on a linear scale with a filter combination consisting of a 450/50-nm bandpass filter (Blue), a 670/30-nm bandpass filter (Red), and a 600-nm longpass filter to split the emission wavelengths. PI emission was measured on a logarithmic scale using a 695/40-nm bandpass filter and 488-nm excitation.

#### 2.2.6. Proliferation

Isolated SP cells were seeded (5 × 10^3^ cells) in 96-well plates in RPMI 1640 without phenol red supplemented with FBS 10%, L-glutamine (2 mM), and gentamicin (50 mg/mL). After 24 h, the medium was replaced and cells were treated with Tx (7.5 and 12.5 µM), doxorubicine (Doxo, 0.1 µM), Fv (0.3 nM), leptin (1000 ng/mL) (R&D systems, Abingdon, UK). Cell proliferation was measured by a resazurin fluorescence test (25 µg/mL) at 24 h post-incubation as described previously. Results were expressed in arbitrary units of fluorescence.

### 2.3. Statistics

Results were expressed as mean +/− SEM. Statistical analysis was performed using the paired, bilateral Student’s t-test, or three-way ANOVA with RStudio, regression with Fisher’s PLSD Post-Hoc test PLSD with StatView^®^ Software (SAS Institute Inc., Cary, NC, USA).

## 3. Results

### 3.1. Study of Breast Cancer Cells

#### 3.1.1. Crosstalk between Mammary Cancer Cells and Adipocytes Is a Key Element in the Resistance of Antiestrogen Therapy and Can Be Mediated by the Indirect Estrogen Pathway

**Figure 1:**
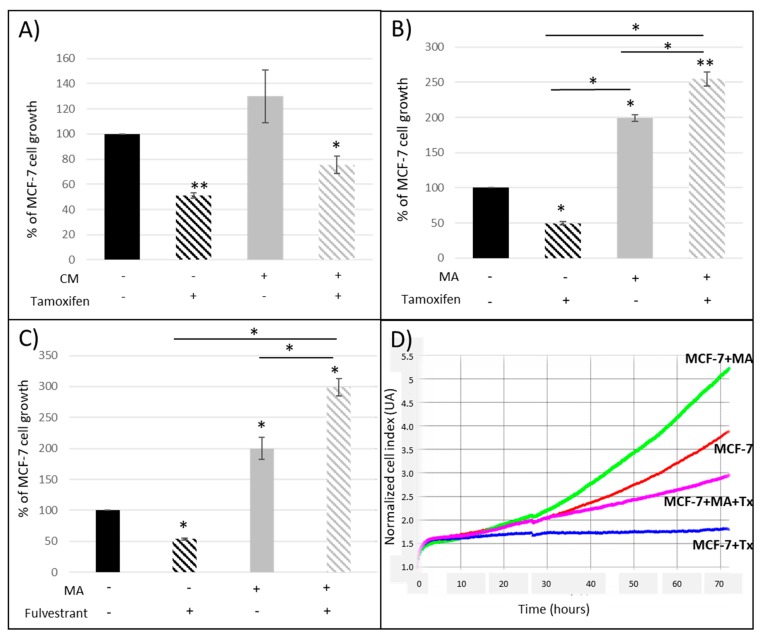
Percentage of MCF-7 cell proliferation when (**A**) cells were cultured with conditioned media (CM) obtained from the culture of human mature adipocytes (MA) and tamoxifen (Tx); (**B**) cells were co-cultured with MA and Tx; (**C**) cells were co-cultured with MA and fulvestrant. (**D**) Representative graph from iCELLigence system. Results are expressed as percentage of MCF-7 cell growth ± SEM at 72 h post-treatment (*n* = 3, * *p* < 0.05, ** *p* < 0.01). [CM: conditioned medium; MA: mature adipocytes].

In the presence of Tx (a selective estrogen receptor modulator (SERM), the proliferation of MCF-7 breast cancer cells was reduced (−50%) (Figure 1A,B), corresponding to expected results since the half-maximal inhibitory concentration was used (IC_50_ = 10 µM) as in our previous experiments [18]. When cancer cells were exposed to adipose secretome through the use of CM (Figure 1A), MCF-7 proliferation was slightly increased (+130%, ns). The antiproliferative effect of Tx was reduced but not significantly (−25% in the presence of CM and Tx vs. −50% with Tx only, ns).

Co-culture experiments were then realized in order to evaluate the interactions between cancer and adipose cells in the presence of antiestrogens (Figure 1B). We found that MA and their secretions were able to double MCF-7 proliferation (+200%, *p* < 0.05 vs. control), to completely abolish the antiproliferative effect of Tx and even to increase the MCF-7 proliferation in the presence of Tx (+254%, *p* < 0.01 vs. control).

These experiments confirmed the role of adipocytes and their secretome, but above all, the crosstalk between adipose and cancer cells.

Similar results were found (Figure 1C) using another antiestrogen agent such as fulvestrant (Fv), a selective estrogen receptor degrader (SERD). The antiproliferative effect of Fv was completely reversed in the presence of adipocytes and their secretions. The proliferation was even increased (+200%, *p* < 0.05), suggesting that adipocytes could also interfere with this pure antiestrogen.

**Figure 2:**
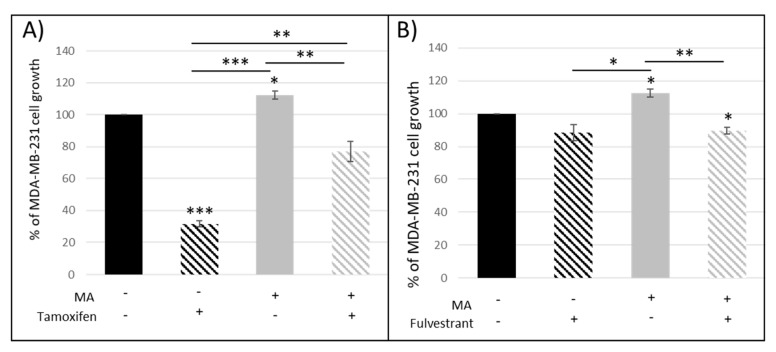
Percentage of MDA-MB-231 cell proliferation when co-cultured with mature adipocytes (MA) in the presence of tamoxifen (**A**) and fulvestrant (**B**) (*n* = 3, * *p* < 0.05, ** *p* < 0.01, *** *p* < 0.001).

Since our results suggested that adipocyte and their secretions could modify the antiproliferative activity of two distinct antiestrogens routinely used in breast cancer treatment, we co-cultured MDA-MB-231, which are ER-mammary cancer cells, with hMAD differentiated cells. Tx was able to decrease the proliferation of these ER-cancer cells (Figure 2A) and this effect was reduced in the presence of mature adipocytes. On the contrary, fulvestrant had no effect on this cell line (Figure 2B). These data suggested that the reduced antiproliferative effect of Tx observed in the presence of adipocytes was mediated by another pathway than the direct estrogen receptor pathway.

Both co-culture and Tx treatment are responsible for specific gene expression profile.

A multiple factor analysis (MFA) was conducted in order to evaluate the balance between two qualitative variable (“co-culture” and “Tx treatment” conditions) and quantitative variables corresponding to gene expression data.

**Figure 3:**
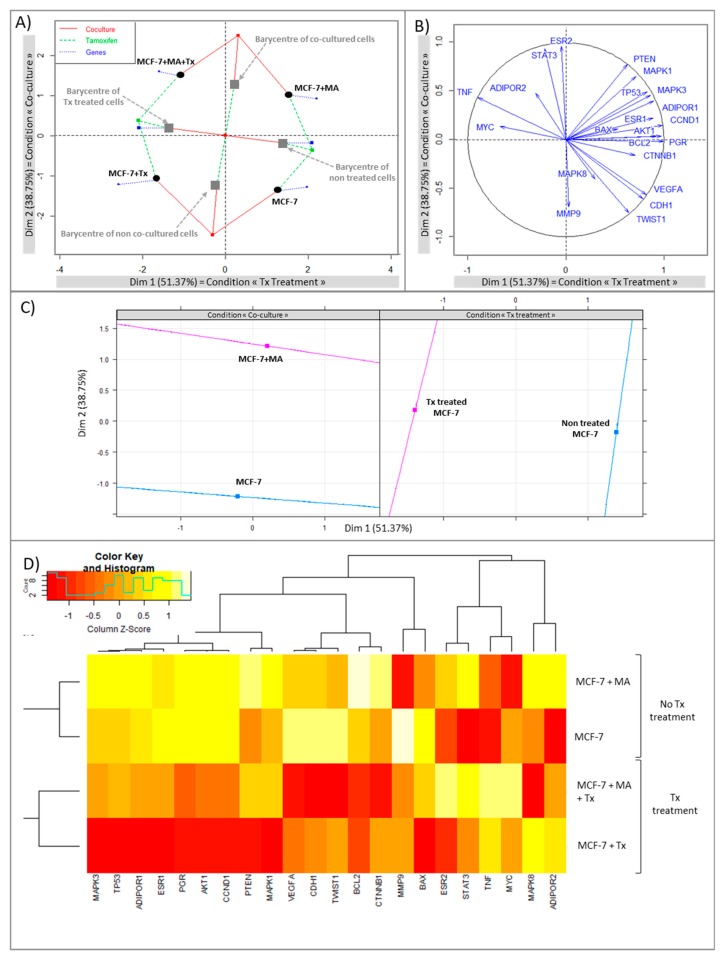
Multiple factor analysis leading to (**A**) individual factor map, (**B**) circle correlation, (**C**) Multiple factor analysis resume, (**D**) heatmap with hierarchical clustering (red for under-expressed, orange for unchanged expression, and white for over-expressed genes). Dimension 1: “Tx Treatment”; Dimension 2: “co-cultured conditions”.

The MFA analysis revealed the most powerful dimension with the condition “Tx treatment” (Dimension 1 explained 51.37% of the variability). The second dimension corresponding to the condition “Co-culture” explained 37.75% of the variability (Figure 3A). Four distinct groups were clearly segregated: control cells; co-cultured cells; cells treated with Tx; co-cultured cells treated with Tx. Barycentres of “Tx treated cells”; and “non treated cells” showed that Tx treatment accentuated the differences between groups according to dimension 1 (green lines). Similarly, barycenters of “co-cultured cells” and “non treated cells” showed that the co-culture accentuated the differences between groups according to dimension 2 (red lines). Blue lines corresponded to the influence of gene expression. An analysis with circle correlation was made (Figure 2B) and a resume of the MFA is supplied in Figure 3C. Hierarchical cluster analysis (Figure 3D) segregated cell types according to Tx treatment which had a high impact on gene expression.

Apoptosis and cell cycle would not be the biological pathways involved in Tx resistance in our experiments.

**Figure 4:**
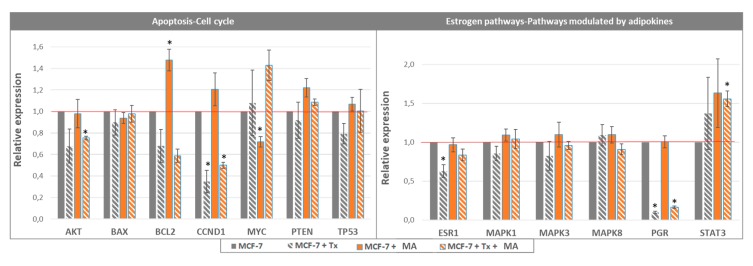
Relative gene expression in MCF-7 cells co-cultured with mature adipocytes and treated with tamoxifen (*n* = 3, * *p* < 0.05).

The analysis with circle correlation (Figure 3B) permitted to observe that Tx treatment was positively correlated with the expression of *MYC* and negatively with *BCL2*, *AKT1*, *CCND1*. Gene expression analysis (Figure 4) showed that the expression of *BCL2*, which was increased in co-cultured MCF-7, was greatly reduced in the presence of Tx (co-cultured MCF-7: relative expression [RE] = 1.48, *p* < 0.05; co-cultured MCF-7 + Tx: RE = 0.49, *p* < 0.05). The expression of *Bax* was not modified between conditions. *Akt* and *CCND1* (cyclin D1) were under-expressed in the presence of Tx (*AKT*: RE = 0.68, *p* = 0.16; *CCND1*: RE = 0.35, *p* < 0.05) and their expression remained stable when cells where cultured with mature adipocytes.

**Figure 5:**
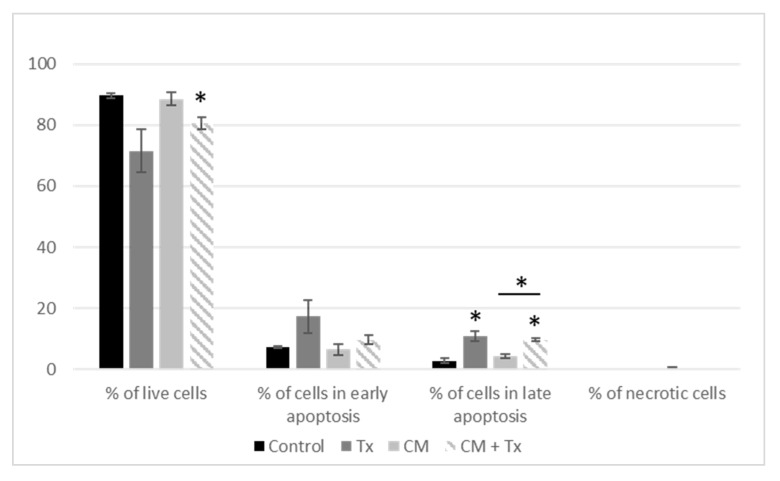
Evaluation of apoptosis after treatment of MCF-7 with conditioned media and tamoxifen. Results are expressed as percentage of MCF-7 cell growth ± SEM at 72 h post-treatment (*n* = 3, * *p* < 0.05). [CM: conditioned medium; Tx: Tamoxifen].

When we evaluated the apoptotic process by an annexin V–FITC/PI apoptosis assay, we found that Tx decreased the percentage of live cells (ns) even in the presence of the adipose secretome (*p* < 0.05) (Figure 5). In parallel, Tx increased the percentage of cells in late apoptosis even in the presence of CM.

#### 3.1.2. STAT3 Could Be a Target in Tx Resistance in the Presence of an Adipose Microenvironment

Concerning the adipokines and the estrogen pathway, the circle correlation (Figure 3B) revealed that the condition “Tx treatment” was positively correlated with the expression of *TNF* and the condition “co-culture” was positively correlated with the expression of *STAT3* and *ESR2*. The evaluation of gene expression (Figure 4) revealed that *ESR1* was significantly under-expressed during Tx exposure, and that this expression returned to a level comparable to that of control in the presence of adipose cells. qPCR analyses also showed a significant increase of *STAT3* expression in the presence of adipose secretome and Tx, suggesting that this pathway may be a target in the drug resistance observed.

#### 3.1.3. Leptin Does Not Seem to Be Involved in the Reduction of the Antiproliferative Effect of Tamoxifen

**Figure 6:**
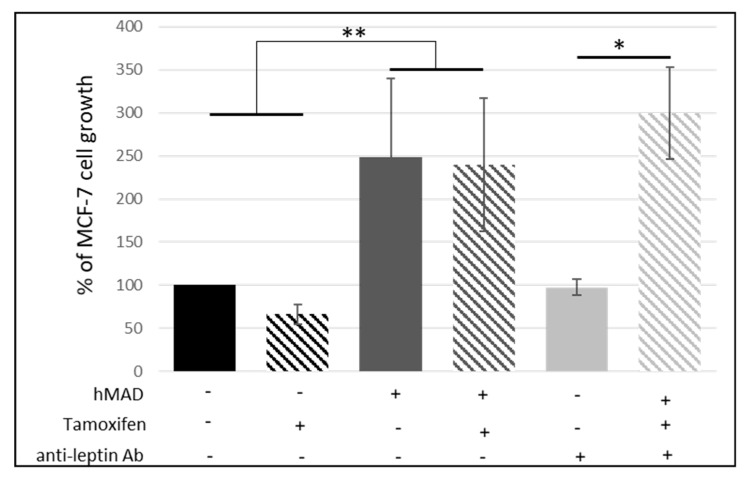
Influence of leptin on anti-estrogen resistance. Percentage of MCF-7 cell growth treated with tamoxifen and anti-leptin antibody (Percentage of growth ± SEM, *n* = 3, * *p* < 0.05, ** *p* < 0.01).

We then investigated if leptin, a major adipokine whose concentrations are increased in obese people, could be involved in the resistance to hormonal therapy. An anti-leptin antibody was used at a concentration of 0.5 µg/mL (according to supplier’s recommendations), which is the highest concentration that we have found in the media, thus neutralizing all the leptin present in our experiments ([leptin] = 3.1 ng/mL in CM). When MCF-7 mammary cancer cells were co-cultured with adipocytes, the anti-leptin antibody had no effect (Figure 6) suggesting that leptin was not the adipokine involved in the decreased antiproliferative effect of Tx. The ANOVA only revealed the discrimination between co-cultured cells vs. non-cultured cells. The analysis of gene expression did not indicate the involvement of leptin, adiponectin, and their receptors in the lower efficiency of Tx.

### 3.2. Study of Cancer Stem Cells (CSCs)

#### Anticancer Treatment and Adipokines Increase the Proliferation of Isolated SP Cells

**Figure 7:**
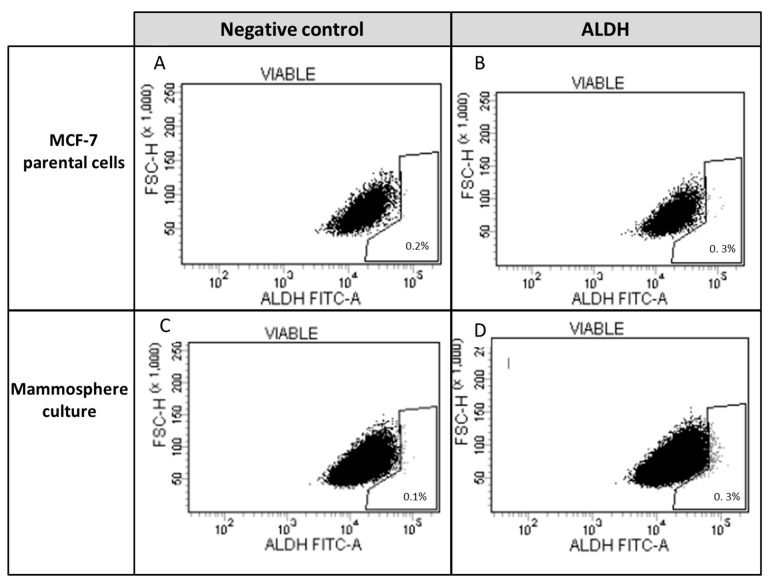
Expression of aldehyde dehydrogenase in parental MCF-7 cells and in mammospheres. Aldehyde dehydrogenase 1+ (ALDH1+) was analyzed by measuring cellular fluorescence of BODIPY-aminoacetate (BAAA) in (**A**) presence or (**B**) absence of DEAB, a specific ALDH1 inhibitor in parental MCF-7 cells and mammospheres, respectively (**C**,**D**). Stem cell fraction is shown in the gate (percent stem cell fraction = percent of ALDH1+ (DEAB−) − percent ALDH1+ (DEAB+)).

The identification of ALDH1+ cells (Figure 7) permitted us to identify 0.1% of ALDH1+ CSCs in parental MCF-7. This number was obtained by subtracting the percentage of ALDH1+ (DEAB+) cells from the percentage of ALDH1+ (DEAB-). When mammospheres were formed after 3 weeks of culture, the number of ALDH1+ cells was doubled in the cell population.

**Figure 8:**
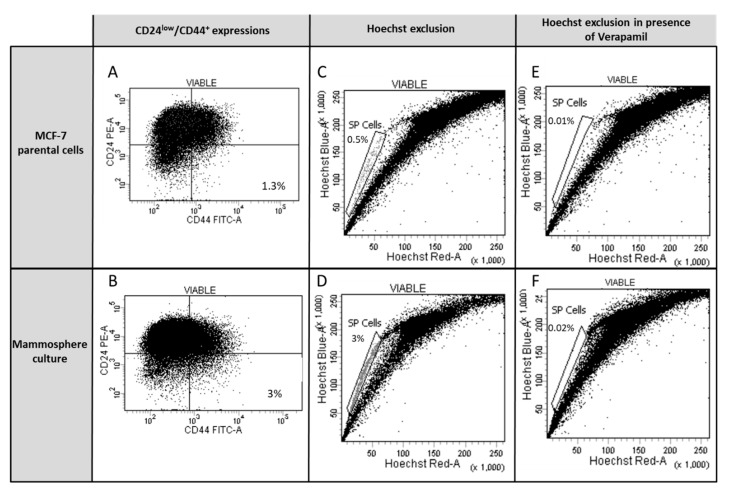
Characterization of the expression of CD24low/CD44+ and the side-population (SP) cells in parental MCF-7 cells and mammospheres. To determine CD24low/CD44+ expression in (**A**) MCF-7 cells and (**B**) mammospheres, cells were incubated with anti-CD24 (PE) and anti-CD44 (FITC) or isotype controls. The SP cells were analyzed without ABC transporter inhibitor Verapamil in (**C**) MCF-7 cells and (**D**) mammospheres, or with 50 µM of Verapamil in (**E**) MCF-7 and (**F**) mammospheres by Hoechst 33342 staining and flow cytometry.

CD24low/CD44+ fraction was similarly increased in mammospheres in comparison with parental MCF-7 cells (from 1.3% in parental MCF-7 cells (Figure 8A) to 3% in mammospheres (Figure 8B)). SP fraction was similarly increased in mammospheres, compared to parental MCF-7 cells (0.5% in parental MCF-7 cells (Figure 8C) to 3% (Figure 8D)). In both MCF-7 (Figure 8E) or mammosphere culture (Figure 8F) conditions, the ABC transporter inhibitor (Verapamil) was able to inhibit Hoechst exclusion, resulting in the disappearance of SP cells in the gate and thus confirming the proper identification of SP cells.

**Figure 9:**
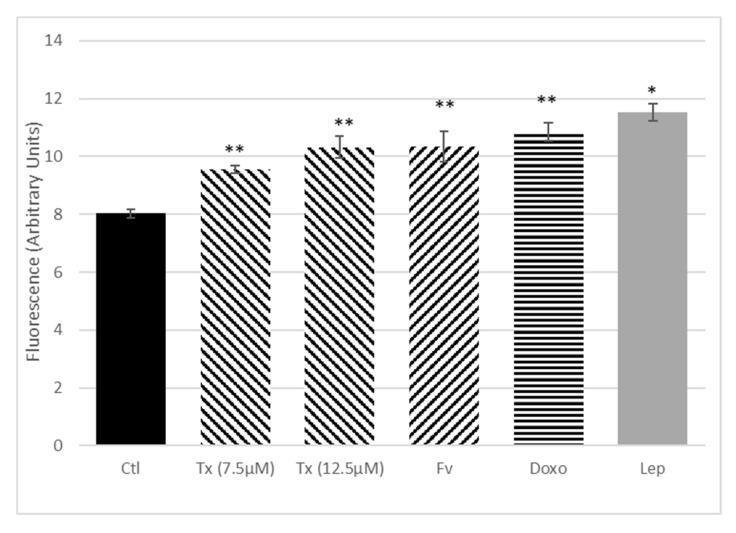
Effect of anticancer treatment, leptin, and Il-6 on the proliferation of isolated side-population (SP) cells. After adhesion, SP cells were treated with different anticancer therapies, i.e., tamoxifen (Tx) at 7.5 and 12.5 µM, fulvestrant (Fv) at 0.3 nM, doxorubicin (Doxo) at 0.1 µM, or with leptin at 1000 ng/mL for 24 h. Cell proliferation was quantified using the resazurin fluorescence test. Bars give means of fluorescence intensity in arbitrary units (AU) ± SEM. Analyses covered three independent experiments; * *p* < 0.05, ** *p* < 0.01, treated vs. control.

The impact of isolated CSCs in anticancer therapy resistance and the influence of adipokines were evaluated on isolated SP cells after exposure to Tx, Fv, Doxo, or leptin. Cell proliferation was measured at 24 h by resazurin assay (Figure 9). All the anticancer molecules used have shown an increase in the SP cell proliferation after 24 h of treatment. In the same way, the SP cell proliferation was also significantly increased after leptin treatment.

## 4. Discussion

Increased BMI and obesity increase breast cancer mortality and some studies highlight the role of the adipose microenvironment in the resistance to cancer treatment [1]. Despite the high efficacy of Tx treatment, a relapse is observed in 10% to 40% of patients depending on initial nodal status and tumor grade [19]. Through tridimensional and co-culture models, we have recently shown that the secretome of adipocytes from obese women is able to reduce the antiproliferative activity of Tx [18]. In this present study, we aimed to identify biological pathways and the involvement of cancer stem cells in the resistance to hormonal treatment.

Using an adipose cell line, which permitted us to eliminate interindividual variations, we confirmed that the adipose secretome and above all, the interactions between adipose and breast cancer cells participated in the lower efficacy of anti-estrogen treatments (Tx and Fv). The mechanisms involved did not seem to be apoptosis and cell cycle but rather the indirect estrogen pathway. This activation may involve adipokines through the activation of the JAK/STAT pathway.

The role of leptin, a major adipokine secreted by adipose tissue, has been largely investigated [20,21] and we have shown that it stimulates the proliferation of breast cancer cells but not that of normal cells [20,22]. The different angiogenic processes (proliferation, migration, and invasion of endothelial cells) are also favored by leptin. In addition, this adipokine is able to reduce the effectiveness of antineoplastic molecules such as 5-fluorouracil, taxol, or vinblastine, as well as anti-estrogens such as Tx [23]. Clinical studies confirmed a positive association between serum leptin levels and breast cancer risk particularly in overweight/obese women [24]. However, our results did not show the involvement of leptin in the resistance of hormonal therapies in our model. We hypothesized that the JAK/STAT pathway could be involved. In the literature, Janus kinases (JAK) and signal transduction and transcription activation (STAT) proteins, particularly STAT3, are described as the most promising targets for cancer treatment. STAT3 is described as both a transcriptional activator and an oncogene that is tightly regulated under physiological conditions. STAT3 is constitutively activated in all breast cancer subtypes and predominantly in triple-negative cancers [25]. A large number of molecules are able to activate the STAT3 pathway. Firstly, cytokines (IL-6, IL-8, IL-11, Oncostatin, IL-10, IL-32), and growth factors are able to bind to receptors with tyrosine kinase activity (RTK) such as EGFR, HER, VEGFR, but also non-RTK (Src) receptors. The activation of STAT3 results in the activation of cell proliferation, survival, invasion, angiogenesis, and also in the epithelial-mesenchymal transition (EMT) [25,26]. Recently, authors highlighted the role of IL-6 in therapeutic resistance [27] and in inducing an EMT-phenotype. By deactivating the secretion of IL-6 by adipocytes and cancer cells, they managed to decrease proliferation, migration, invasion, and EMT, suggesting the paracrine role of this adipokine [28]. Thus, the different actors of the mammary adipose microenvironment and the cancer cells themselves can activate the STAT3 pathway in an autocrine or paracrine manner.

A large proteomic study of adipose tissue samples collected next to mammary tumors identify a large number of hormones, cytokines, and growth factors involved in various biological pathways such as signal transduction, cell growth, immune response, or apoptosis [29]. Recently, conditioned media of mammary adipose tissue from women with breast cancer have been shown to promote proliferation, adhesion, and migration of mammary epithelial cells, contrary to the conditioned media from the adipose tissue of non-cancer patients, suggesting the importance of soluble adipose tissue factors nearby tumor cells [30]. In addition, mammary cancer cells may alter the phenotype of adipocytes, which in turn would promote tumor aggressiveness and local invasion [31]. These adipocytes located near the tumor are called “cancer-associated adipocytes” (CAA) and are characterized by a loss of lipid content, a decrease in late marker expression of adipogenesis, and an overexpression of inflammation markers (IL-6, IL-1β) and proteases (MMP-11, PAI-1) [31]. A recent study confirmed that CAA are smaller than adipocytes located more than 2 cm from the tumor or adipocytes from a breast without tumor. Moreover, it seems that these adipocytes more strongly express the versican (proteoglycan involved in the binding of cells with the extracellular matrix), AdipoR1 (compensatory phenomenon following the decrease of the expression of adiponectin), and CD44 (role in the adhesion and migration), and on the contrary show a decrease in the expression of adiponectin and perilipin (MA marker) [32].

We had previously shown the role of leptin in interfering with anti-cancer treatments [23], and herein we wanted to see if leptin had any activity on cancer stem cells (CSCs). Indeed, there is emerging evidence that CSCs are involved in breast cancerogenesis and resistance to anticancer therapies in breast cancer. However, the low number of these cells makes their analyses and functionality difficult to study. Under these conditions, mammosphere formation increased the percentage of SP cells, ALDH1^+^, and CD24^low^/CD44^+^ fractions and offered a better way to determine the functionality of these cells. Our results on the treatment of isolated SP fractions showed that CSCs were resistant to anticancer treatment such as Tx, Fv, and doxorubicin. It was recently found that Tx had the ability to promote the survival of cells with stem cell properties in MCF-7 cells, since a pretreatment with 4-OH-Tx raised the ability of these cells to form mammospheres [15]. Conversely, Ao et al. showed that treatment with 4-OH-Tx or Fv induced a decrease in cell proliferation, even if 4-OH-Tx or Fv did not affect the ability of these cells to form mammospheres or their tumorigenicity [15]. The difference between the two studies was explained by the differences in concentrations of 4-OH-Tx used and also in technical design. Our results also indicated that leptin was able to increase SP cell proliferation. It was previously shown that leptin was involved in the stimulation and maintenance of breast CSCs [33] and that CSCs survival, evaluated through mammosphere formation, increased in response to leptin, along with an increase in leptin receptor gene expression [34]. Adipokines such as leptin could therefore be involved in CSCs-induced resistance to anti-cancer treatment, especially in overweight patients.

Thus, adipose secretome and cellular interactions seem to play a key role in resistance to hormonal therapy. This resistance cannot be explained by a single process. Among the pathophysiological mechanisms involved, leptin does not seem to interfere with the estrogenic pathway but could favor the proliferation of CSCs. Further studies are needed to identify targets and facilitate the implementation of personalized care for overweight breast cancer patients, which should be considered as an unique patient population.

## Abbreviations

CM	Conditioned media
ER	Estrogen receptor
Fv	Fulvestrant
MA	Mature adipocyte
Tx	Tamoxifen
